# Supplementary material for: Turnover of the extracellular polymeric matrix of granules performing biological phosphate removal
Source: Appl Microbiol Biotechnol. 2023 Feb 10;107(5-6):1997–2009. doi: 10.1007/s00253-023-12421-7 (PMC10006046; doi:10.1007/s00253-023-12421-7)
Supplement: Supplementary file 1 — Fig. S1 Detailed microbial community composition. Relative genus-level microbial community distribution based on 16S rRNA gene amplicon sequencing (AmpSeq) and metaproteomics (MetProt) based on identified peptides. For AmpSeq, all OTUs contributing < 1 % are grouped as “Others”. For MetProt, all the groups cointributign < 0.5 % are represented by “Others” (PDF 203 kb) [file 253_2023_12421_MOESM1_ESM.pdf]

Applied Microbiology and Biotechnology

Supplementary Figure

Title: Turnover of the extracellular polymeric matrix of granules performing biological phosphate removal

Sergio Tomás-Martínez*[1], Erwin J. Zwolsman[1], Franck Merlier[2], Martin Pabst[1], Yuemei Lin[1], Mark C.M. van Loosdrecht[1], David G. Weissbrodt [1]

1 Department of Biotechnology, Delft University of Technology. Van der Maasweg 9,2629 HZ, Delft, The Netherlands

2 CNRS Enzyme and Cell Engineering Laboratory, Université de Technologie de Compiègne, Rue du Docteur Schweitzer, CS 60319, 60203 Compiègne Cedex, France

*Corresponding Author: Sergio Tomás-Martínez

Address: Department of Biotechnology, Delft University of Technology. Van der Maasweg 9,2629 HZ, Delft, The Netherlands

E-Mail: S.TomasMartinez@tudelft.nl

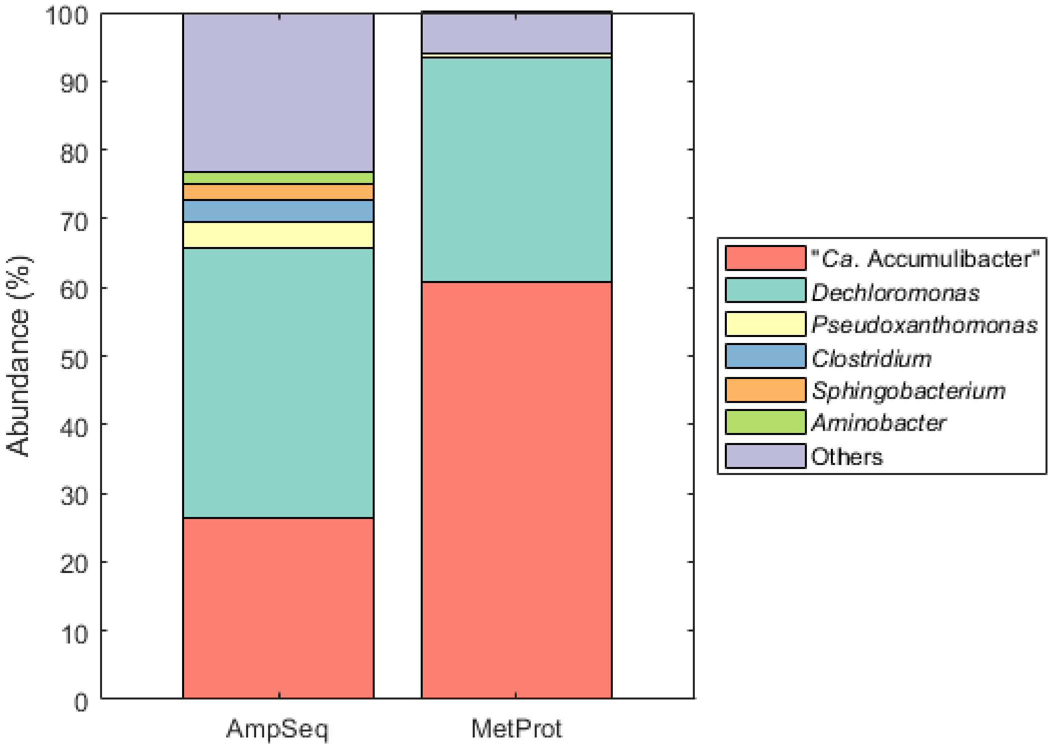

Fig. S1 Detailed microbial community composition. Relative genus-level microbial community distribution based on 16S rRNA gene amplicon sequencing (AmpSeq) and metaproteomics (MetProt) based on identified peptides. For AmpSeq, all OTUs contributing < 1 % are grouped as “Others”. For MetProt, all the groups cointributign < 0.5 % are represented by “Others”
